# Arthroscopic-assisted radiocarpal ligaments tensioning for dynamic radiocarpal instability

**DOI:** 10.1186/s12891-021-04857-7

**Published:** 2022-02-17

**Authors:** Wei-Chen Hung, Jung-Pan Wang, Yi-Chao Huang, Cheng-Yu Yin, Cheng-Yi Wu, Hui-Kuang Huang

**Affiliations:** 1 Department of Orthopaedics, Ditmanson Medical Foundation Chiayi Christian Hospital, Chiayi, Taiwan; 2 Department of Orthopaedics & Traumatology, Taipei Veterans General Hospital, Taipei, Taiwan; 3 Department of Surgery, School of Medicine, National Yang Ming Chiao Tung University, Taipei, Taiwan; 4 Department of Food Nutrition, Chung Hwa University of Medical Technology, Tainan, Taiwan

**Keywords:** Arthroscope, Dynamic radiocarpal instability, Radioscaphocapitate ligament, Tensioning, Wrist

## Abstract

**Background:**

Dynamic radiocarpal instability is one of the causes of post-trauma radial-sided wrist pain. It is not easy to diagnose and may possibly be overlooked. The key ligaments responsible for dynamic radiocarpal instability are the radioscaphocapitate (RSC) and long radiolunate (LRL) ligaments. Tensioning of these 2 ligaments could be a method of treatment for dynamic carpal instability. We proposed a method for arthroscopic thermal shrinkage of these 2 ligaments, and for setting a landmark arthroscopically to facilitate identification of these 2 ligaments during the combined open suture tensioning procedure.

**Methods:**

Between January 2016 and May 2020, 12 patients treated with this method were enrolled. The mean age was 33.3 years (range, 18–57 years), and the mean duration from injury to operation was 7.8 months (range, 3–25 months). The diagnosis was mainly depended on the physical examinations and confirmed under arthroscopy. The mean follow-up was 17.7 months (range, 12–26 months).

**Results:**

All the patients had marked improvement of pain, grip strength, the Disabilities of the Arm, Shoulder and Hand questionnaire (DASH), and the radiocarpal stability. The wrist range of motion showed significant decrease around 5^o^ in both flexion and extension and around 4^o^ in the ulnar deviation at the final follow-ups. All patients were able to return to their previous full level of work and activities.

**Conclusions:**

We conclude that arthroscopic thermal shrinkage combined with open suture tensioning can be effective in treating dynamic carpal instability, while the arthroscopic-assisted landmark setting can help identify the accurate location of the RSC and LRL ligaments without dissecting too much soft tissue.

## Background

Post-traumatic radial-sided wrist pain is a common issue in clinical practice [1, 2]. Fractures or dislocations can be identified on radiographs or in other detailed imaging examinations. Mayfield described a mechanism to explain the injury force transmission from the radial to ulnar wrist ligaments during traumatic injuries [3]. The radioscaphocapitate (RSC) and long radiolunate (LRL) ligaments are mainly responsible for radial-sided radiocarpal stability. Injuries to these two ligaments could lead to carpal ulnar translation or carpal instability nondissociative (CIND) [4–7]. However, these radio-volar ligaments are occasionally only partially injured, and the translation of the carpus may not be observed. Nevertheless, the injured ligaments would not be stable enough to maintain radial-side radiocarpal stability, and therefore, cause dynamic radiocarpal instability, which in turn causes symptoms, such as radial wrist pain, weakness, and clicking sounds during wrist activity [2].

A diagnosis of dynamic radiocarpal instability is difficult because image examination may not provide enough information. The main diagnostic method is a physical examination [2, 8]. Regarding treatment, ligament tensioning can be effective if the diagnosis can be confirmed [2]. However, accurate localisation of the key ligaments for open tensioning treatment would be difficult because the border of the outer layers cannot be easily defined, especially in scarred conditions after injury.

Here, we present an arthroscopic treatment algorithm for the treatment of dynamic radiocarpal instability. It includes arthroscopic thermal shrinkage of the radio-volar ligaments and marking of these ligaments to facilitate combined open suture tensioning.

## Methods

The study design was approved by the Institutional Review Board of our institute. All methods were carried out in accordance with relevant guidelines and regulations.

### Study protocol

A fall to an outstretched hand is a common cause of wrist injury. With radial wrist pain, initial standard radiographs of the wrist and hand are taken to rule out possible bony or ligamentous injuries. If the patient presents with tenderness around the scaphoid, a scaphoid fracture is initially suspected. If plain radiographs do not reveal a significant fracture of the scaphoid or around the radial wrist, a detailed imaging examination is warranted.

**Fig. 1:**
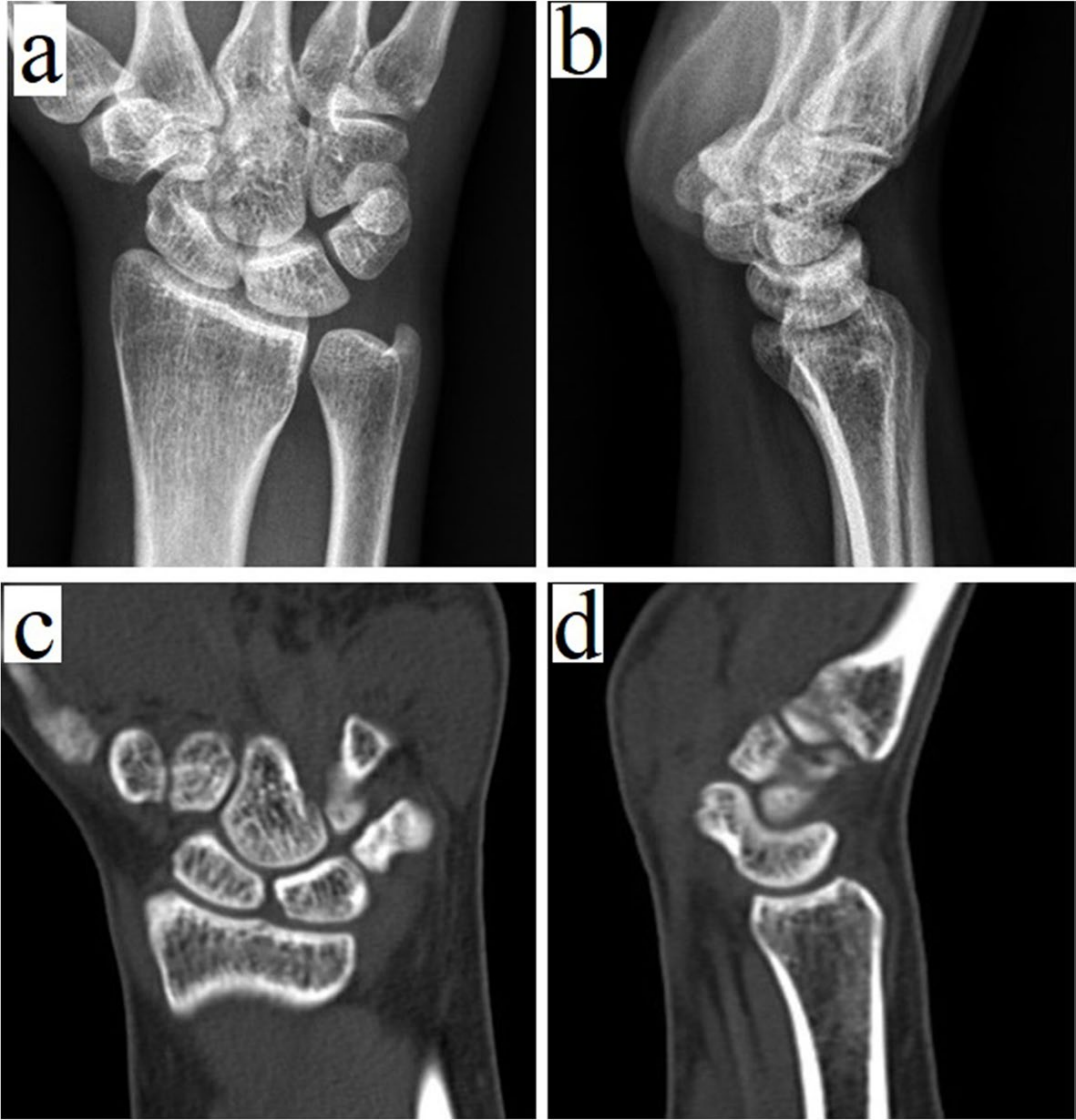
A 29-year-old female. (**a**, **b**) Radiographs and (**c**, **d**) compued tomography of her left wrist 11 months after the injury event showed no identifiable abnormality of the wrist structure

A stability test would not be accurate enough to reveal the instability of the wrist in an acute state after injury due to wrist pain and swelling. In addition to the routine radiographs of the wrist, we prefer to perform a computed tomography (CT) scan of the wrist if any carpal occult fracture or any undetected fracture is still suspected (Fig. 1). If no definite fracture can be identified on CT, physical examination strongly indicates a significant injury, repeated physical examinations and radiographs during the period of conservative treatment are important [9, 10]. Attention should be paid to the possibility of undetectable scaphoid occult fracture in the initial CT images or the possible transformation of scaphoid bone bruising to occult fracture. The immobilisation of the wrist would be a helpful treatment in the conservative treatment period to help control swelling and reduce pain.

During follow-ups, physical examinations play an important role in the diagnosis of instability. Tenderness around the scaphoid, especially at the volar aspect, would be a hint of dynamic instability.

Patients with dynamic radiocarpal instability may complain about pain and weakness during activity, and sometimes even at rest. Clicking during wrist motion may possibly be observed. During the scaphoid shift test by applying pressure on the patient’s scaphoid tubercle and moving the patient’s wrist from ulnar to radial deviation, subluxation of the scaphoid and elicitation of pain may be noted [11]. In addition, a carpal stability test is performed by holding the scaphoid between the examiner’s thumb and index fingers and moving in the dorsal and volar directions relative to the radius. Pain and increased laxity compared to the healthy contralateral side would be noted in patients with dynamic radiocarpal instability.

### Indications

1. Patients with dynamic radiocarpal instability, presenting with radial wrist pain and a positive carpal stability test showing apparent laxity compared to the other side.
2. Patients with RSC and LRL ligamentous laxity without apparent tears or avulsions.

### Contraindications

1. Patients with immunologic diseases, such as rheumatoid arthritis, should be excluded because the ligamentous pathogen is generally around the wrist and not in the specific RSC and LRL ligaments.
2. Patients with apparent carpal ulnar translation as they should be suitable for undergoing ligament reconstruction or a focal arthrodesis procedure.
3. Patients with wrist degeneration as they should be suitable for undergoing wrist total/focal arthrodesis or other salvage procedures.
4. Patients with radial-sided pain from other causes, such as scaphoid or other wrist fractures, tenosynovitis, or an acute stage of soft tissue swelling.

### Surgical techniques

We prefer that patients undergo under general anaesthesia while performing arthroscopy on the patient’s wrist in a relaxed state and use an arm pneumatic tourniquet.

Patients with dynamic carpal instability would have a lax wrist so that the scaphoid may be displaced over the scaphoid fossa if the wrist is held in a semi-flexed position in the traction tower. A displaced scaphoid would make it difficult to insert the arthroscope into the 3–4 viewing portal. Manually pushing the carpal bones backwards could normalise the radiocarpal alignment in the anteroposterior direction to facilitate the arthroscope entering the radiocarpal space.

During arthroscopy, the intraarticular structures of the proximal and mid-carpal joints should be checked with a setup of standard 3–4, 6R, and midcarpal (radial and ulnar) portals. The concept of the Mayfield injury mechanism indicates the force of injury from the radial to the ulnar side of the wrist. Therefore, the RSC/LRL and scapholunate (SL) joints should be carefully checked to identify any tear injuries of the RSC/LRL or SL interosseous ligaments. The use of a probe to hook the SL interval to check its tightness and evaluate the laxity of the RSC/LRL ligaments is helpful. If there is any significant SL dissociation, it can be identified during previous image studies and physical evaluations.

In case of findings of lax RSC and LRL ligaments, thermal shrinkage should be performed. It is appropriate to set the 4–5 portal as the viewing portal and the thermal probe in the 3–4 (or 1–2) portal. A thermal tensioning of the RSC and LRL ligaments in the radiocarpal joint is then performed. In the midcarpal joints, the thermal tensioning of the RSC midcarpal portion, as well as the volar wrist capsule, is also performed. An Oratec Micro-TAC-S probe (Oratec Interventions, Menlo Park, CA) for thermal management, with the energy set at 67 °C and 40 W is used. The probe is gently swept over the ligaments. After thermal shrinkage, a switch stick into the interval between the RSC and LRL ligaments is inserted. The switch stick bluntly protrudes toward the skin. After ensuing that the switch stick is directly against the skin without any interposed soft tissue structures, an incision is made in the skin at a position tented by the stick to allow the stick to come out of the skin.

**Fig. 2:**
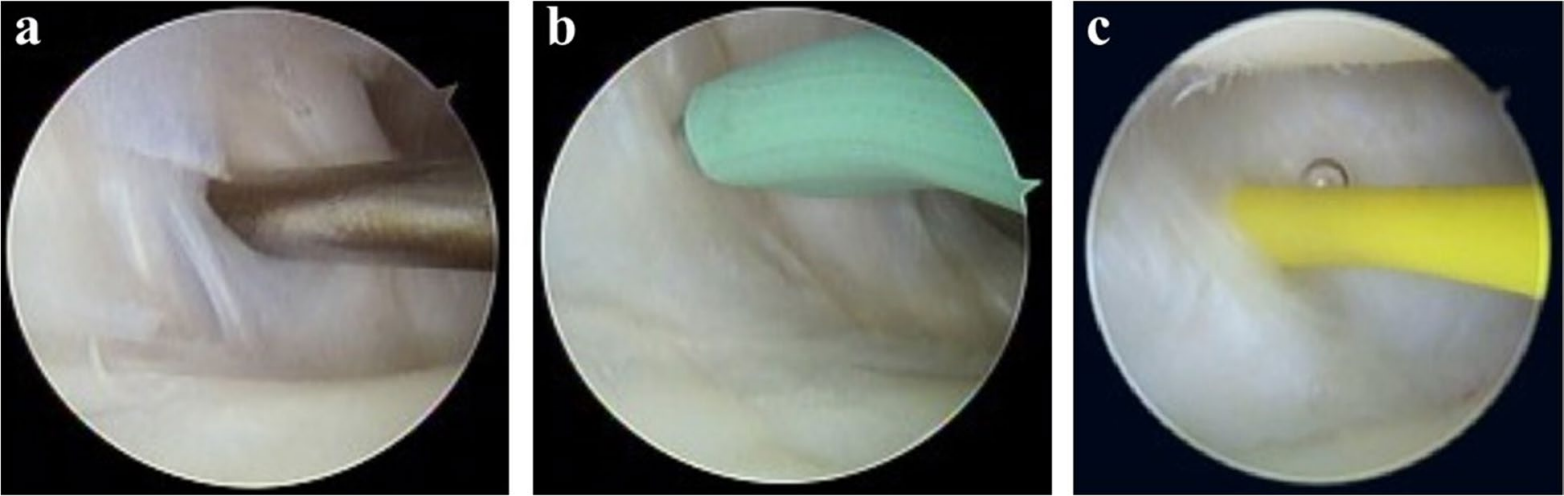
**a** The radioscaphocapitate (RSC) and long radiolunate (LRL) ligaments of the patient’s left wrist were hooked by the probe, showing apparent laxity, **b** Arthroscopic thermal shrinkage for the RSC and LRL ligaments, **c** Setting the vessel loop inside-out to mark the interval of the RSC (right) and LRL (left) ligaments

**Fig. 3:**
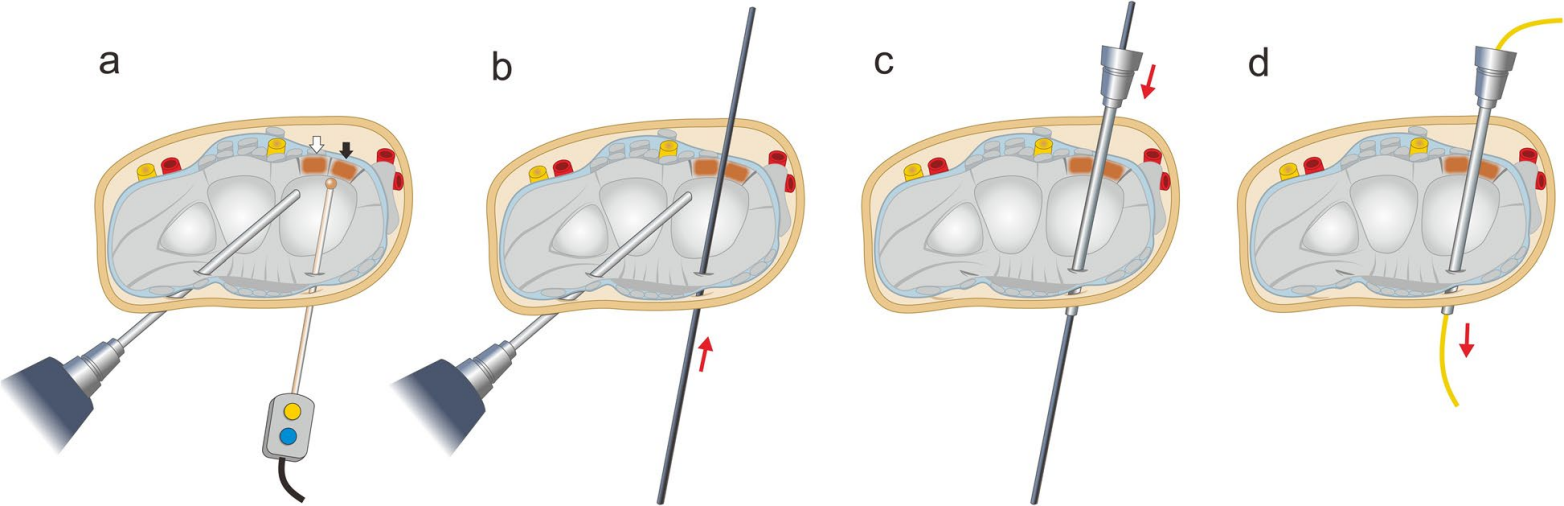
Schematic diagram showing the steps of arthroscopic procedures. **a** Perform thermal shrinkage for the radioscaphocapitate (RSC) (black arrow) and long radiolunate (LRL) (white arrow) ligaments from 3 to 4 (or 1–2) portal. **b** Inset a switch stick into the interval between the RSC and LRL ligaments and forward it volarly to come out of the volar wrist skin. **c** Set the arthroscope sheath onto the switch stick from the volar side and forward it dorsally to come out of the dorsal wrist skin. **d** Removal of the switch stick and subsequently set the vessel loop through the canal of the arthroscopic sheath

The arthroscope sheath is set onto the switch stick from the volar side, and directed into the radiocarpal joint along the switch stick. It is then forwarded dorsally until the sheath comes out of the dorsal wrist skin. Subsequently, the switch stick is removed, and a string of vessel loops from the arthroscope sheath opening on the dorsal wrist id insert. The vessel loop is passed through the sheath canal to exit the sheath opening on the volar wrist. The sheath is removed, and the vessel loop is retained with its two sides protruding from both the volar and dorsal wrist skin. The route of the vessel loop indicates the interval between the RSC and LRL ligaments (Figs. 2 and 3).

The traction force is then released, and carpal stability is tested. If stability is improved to a level similar to that of the contralateral healthy side, the vessel loop is removed and the tensioning procedures are completed.

**Fig. 4:**
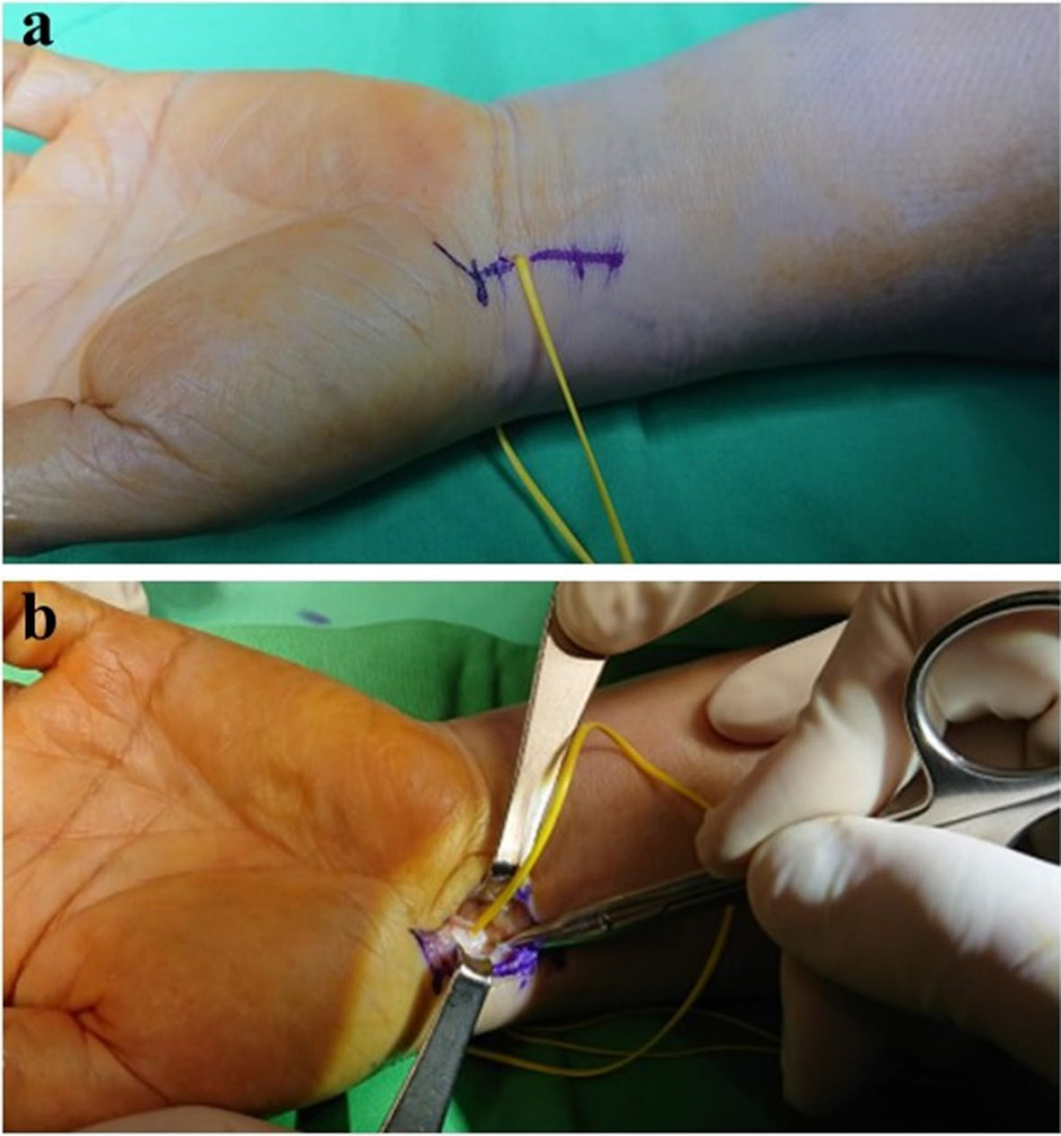
**a** Illustrating the open approach along the mark (vessel loop), making access to the radioscaphocapitate (RSC) and long radiolunate (LRL) ligaments easy and precise. **b** The vessel loop indicates the interval of the left wrist RSC and LRL ligaments (left wrist)

If the test still shows radiocarpal instability or the patients perform high-demand hand labour, an open tensioning surgery could be performed. To perform open tensioning, we prefer using a zigzag incision over the flexor carpi radialis (FCR), centred on the volar vessel loop tail (Fig. 4). The dissection would be very straightforward, where a deeper incision along the vessel loop is made as the FCR is retracted ulnarly, ensuring not to injure the radial artery. Opening the FCR tendon sheath would be helpful in providing a good and wide operative field. The vessel loop should penetrate deep into the periarticular fat tissue, and pushing away these fat tissues around the vessel loop with a Freer elevator would be of help to reveal the ligamento-capsule structure. The RSC ligament is on the radial-distal side of the vessel loop, and the LRL ligament is on the ulnar-proximal side. The use of the probe or a Freer elevator for palpation can help in detecting the outer margin of the RSC and LRL ligaments and the space of Poirier, which is toward the distal part of the RSC/LRL interval.

**Fig. 5:**
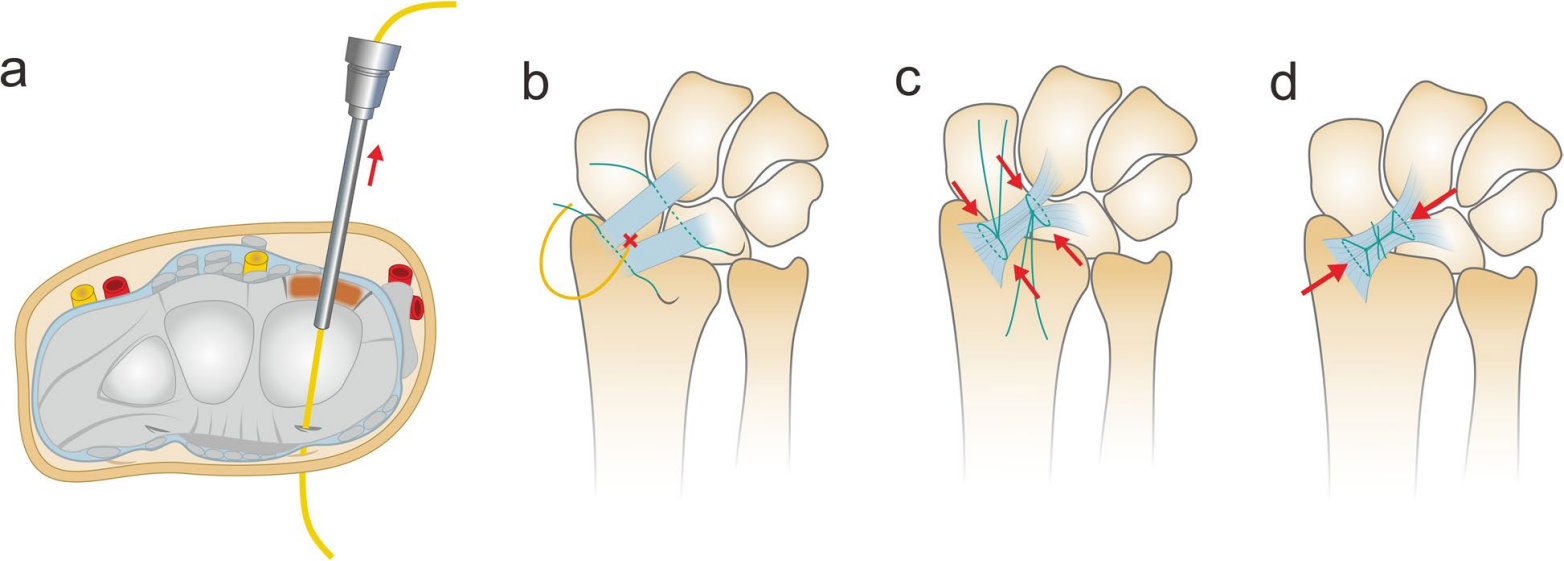
Schematic diagram showing the steps of open suture tightening. **a** The setting of the vessel loop through the interval of the radioscaphocapitate (RSC) and long radiolunate (LRL) ligaments. **b**,**c** The sutures were tightened to close the RSC/LRL interval and the space of Poirier. **d** Thereafter, the suture tails of both ties were tightened together to tense the RSC and LRL ligaments

We use 2–0 non-absorbable (Ethibond 2–0; Ethicon USA) sutures, with one suturing both the RSC and LRL ligaments together proximal to the vessel loop and another suturing the two ligaments distal to the vessel loop. The two sutures are tied separately to tighten and close the interval of the RSC and LRL ligaments and the space of Poirier. Thereafter, the suture strings of the two ties are tied together to tighten and tension the RSC and LRL ligaments (Fig. 5). After the suture tensioning procedure, the stability is checked, and the radiocarpal instability can be improved. When closing the wound, if the FCR sheath is open, it is repaired with absorbable sutures. The skin is then closed using 4–0 nylon sutures.

Below are some guidelines for the surgical techniques used in this study.

1. In the open approach for suture tensioning, the vessel loop is set between the RSC and LRL ligaments. However, the outlying margin is difficult to identify. The use of the probe helps to identify the direction of the ligament fibres and the general margin. There is also a risk of injury to the RSC and LRL ligaments if the surgeon tries to remove the fascia tissue after the periarticular fat tissues have been removed.
2. The direction of the RSC and LRL ligaments should be more parallel to the wrist crease direction than to the forearm axis from the perspective of the open wound field. Therefore, the RSC ligament should be on the radio-distal side of the vessel loop and the LRL ligament on the ulnar-proximal side of the vessel loop.
3. There is a mid-carpal portion of the RSC ligament, and the space of Poirier is also located close to it. As the proximal suture is just proximal to the vessel loop, which comes out from the radiocarpal joint, the distal suture should be further away from the vessel loop to grasp the mid-carpal portion of the RSC ligament entity.

### Postoperative management

After surgery, a short-arm splint with the wrist at 0^o^ of flexion was used for 6 weeks. Subsequently, gentle wrist range of motion (ROM) exercises were initiated. Strengthening and gradual return to normal activities were allowed 3 months after surgery.

### Postoperative assessment

Follow-ups were arranged once every 2 weeks for the first month after surgery, and once every month thereafter for at least 4 months. Additional follow-ups were performed, if indicated. A follow-up 6 months later and yearly thereafter was then conducted.

Functional outcomes were evaluated during follow-ups beginning at 3 months postoperatively. During that time, the scaphoid shift test was performed, and the radiocarpal joint stability was checked. Pain level was evaluated using the visual analogue scale (VAS) for pain (0, no pain; 10, worst pain) at rest, during activity, and active ROM. The Disabilities of the Arm, Shoulder, and Hand questionnaire (DASH) score [12] and the returned level of activity were recorded based on a self-reported questionnaire.

### Statistical evaluations

The pre- and postoperative functional results were compared and analysed using the Wilcoxon signed-rank test. The significance level was set at *p* < 0.05. All statistical analyses were performed using SPSS software (IBM SPSS Statistics for Windows. Version 24.0. Armonk, NY: IBM Corp, 2016).

## Results

We reviewed patients who had dynamic radiocarpal instability and underwent arthroscopic-assisted thermal shrinkage combined with open ligament suture tensioning between January 2016 and May 2020. All enrolled patients were noted to have no significant structural injuries based on CT and/or magnetic resonance imaging. In total, we included 12 patients (12 wrists; 4 men and 8 women). The cause of injury in 11 patients was a fall on the outstretched hand, and one patient experienced the injury when using the outstretched hand to support a heavy falling object. All patients had undergone conservative treatment for at least 3 months after the injury and had persistent symptoms of radial-sided wrist pain and/or clicking sounds during activity. All patients had a positive carpal stability test result, indicating apparent laxity compared to the other healthy side. No carpal ulnar translation was noted on radiographs of the 12 patients. In the arthroscopic finding, all the patients were noted to have RSC/LRL ligament laxity, as tested by hooking with a probe.

The mean age of the 12 patients was 33.3 years (range, 18–57 years) and the mean duration from injury to operation was 7.8 months (range, 3–25 months). The mean follow-up period was 17.7 months (range, 12–26 months). Compared with the patients’ preoperative status, the mean functional results of pain, grip strength, and DASH scores had markedly improved at the final follow-up. The average wrist ROM showed a significant decrease in both flexions (from 75.0^o^ to 70.4^o^) and extension (from 80.4^o^ to 74.6^o^) and ulnar deviation (from 35.4^o^ to 31.3^o^) (Table 1).

**Table 1:** Functional results of the 12 patients before surgery and at the final follow-ups

	Pre-operation Mean (SD)	Post-operation Mean (SD)	*P*-value
Range of motion (degree)			
Flexion	75.0 (6.7)	70.4 (5.4)	0.016
Extension	80.4 (5.4)	74.6 (6.6)	0.006
Radial deviation	23.3 (2.5)	22.5 (2.6)	0.414
Ulnar deviation	35.4 (4.5)	31.3 (4.3)	0.004
VAS at rest	1.8 (0.5)	0 (0)	0.002
VAS during activity	5.9 (1.0)	1.3 (0.8)	0.002
Grip strength (Kg)	14.6 (4.2)	27.0 (4.6)	0.002
DASH score	53.2 (7.3)	14.3 (5.1)	0.002

*SD* Standard deviation

All patients could return to their previous level of work and activities, while 3 patients needed to wear a wrist brace for heavy-loading activities. Improved radiocarpal stability was maintained in all patients at the final follow-up. There were no minor or major complications, and the final radiographs showed no carpal translation and no apparent degenerative changes.

## Discussion

The proposed method used a wrist arthroscope to facilitate the treatment of dynamic radiocarpal instability. An arthroscope was used to perform thermal shrinkage for the RSC/LRL ligaments, as well as for the volar wrist capsule. The arthroscope assisted in setting a mark for the RSC and LRL interval and helped in providing easy access to the targeted ligaments from an open approach for suture tensioning.

In the arthroscopic portals set up, we suggest that any difficulty in inserting the arthroscopic cannula into the 3–4 portal is a hint to reconfirm the diagnosis of radiocarpal instability when the wrist is in the semiflexion position in the traction tower and the carpal bones are pushed backwards to facilitate normalising the scope cannula insertion. In addition, the scaphoid displaced out of the scaphoid fossa is also a sign of radiocarpal instability [2].

Arthroscopic thermal shrinkage was first introduced for shoulder instability with good short-term results and has since been widely practised. However, good results cannot be sustained in the long term [13, 14]. Studies on thermal shrinkage of the capsule or ligaments in wrist instability, mainly for mid-carpal instability, have also shown good results in short- and mid-term follow-ups [15, 16]. In the study by Hargreaves, follow-ups longer than 10 years showed 80% excellent results in the same cohort, with no significant deterioration over time [17]. It was considered that the wrist could be reinforced with postoperative immobilisation to enhance the effect of thermal shrinkage and ligamentous shortening during immobilisation, in a way that could not be performed with the shoulder, elbow, and finger joints [18–22]. Dynamic radiocarpal instability is likely to involve limited injury to the radiocarpal ligaments and mainly the RSC and LRL ligaments. As thermal treatment was reported to be effective for the complex and broadly involved ligaments in midcarpal instability [15, 16], the same thermal treatment for these ligaments with focused laxity is promising.

This method is similar to that used by Rubensson et al. for treating dynamic radiocarpal instability [2]. They proposed a method of dividing the ulnar half of the RSC from its proximal insertion and passing it through a split in the mid-substance of the LRL and then suturing it back to the radial half of the RSC ligament. This method aims to shorten half of the RSC ligament and close the space of Poirier. Their results showed that 15 of 18 patients who were assessed during the postoperative mean 2.5 years were symptom-free or markedly improved, and 12 of 14 patients who could be followed up for 11–15 years were symptom-free or had markedly improved. The results showed that tensioning of the RSC (as well as the LRL) ligament and closing the space of the Poirier were beneficial for treating problems due to injured RSC and LRL ligaments that were in a stretched and increased laxity status. This technique is also effective against dorsal translation of the scaphoid, which occurs when the scaphoid-lunate biomechanical unit is damaged [23–25].

Capsuloligamentous plication or tightening with an open approach for midcarpal or radiocarpal instability is an effective method [26]. Therefore, we believe that open extraarticular capsuloligamentous plication for injured and laxed RSC and LRL ligaments, in addition to intra-articular thermal shrinkage, would help in increasing radiocarpal stability. Regarding the anatomic position, suturing the RSC and LRL ligaments together to make them close could reinforce the anterior complex of the SL ligament. This may be helpful if SL interosseous ligament injury coexists.

In our proposed method, there is no need to demark the margin of the RSC and LRL ligaments, which may injure these ligaments. With the landmark of the vessel loop to indicate the interval of the RSC and LRL ligaments at the radiocarpal joint level, the suture can grasp the RSC and LRL ligaments together with the capsules to achieve capsuloligamentous plication.

Regarding the reduction of ROM of the wrist, the two effects of wrist tightening from arthroscopic thermal tensioning and open suture capsuloligamentous tensioning must be considered. In the reviews mentioned, the use of thermal shrinkage in treating midcarpal instability could maintain most of the wrist motion. In the Mason and Hargreaves results, of 9 patients with a minimum of 14-month follow-up, a global reduction in the wrist ROM of 8^o^ (15%) was noted [16]. In a study by Carin Rubensson et al. on open tensioning of the RSC and LRL ligaments in treating dynamic radiocarpal instability with a mean follow-up of 2.5 years [2], 9 out of 18 patients showed complete recovery of mobility compared to the uninjured side. Our results also showed only a mild reduction in wrist motion at the final follow-up compared to the preoperative status. We believe that arthroscopic minimally invasive treatment that addresses only the targeted radiovolar ligaments could minimise soft tissue dissection, even with the open approach, since the target suture location is already marked. Less scar tissue would lead to less reduction in wrist ROM.

We did not treat patients with ulnar translation of the carpus with this method; therefore, we do not suggest adapting this method as a treatment for these patients, as this method is used mainly for tensioning ligaments. If carpal translation appears, it should indicate that the ligaments are in a severely injured condition and tensioning could not make the ligaments strong enough to hold the carpus on the radius oblique platform. Therefore, in the translated carpus, ligament reconstruction or limited wrist fusion, such as radiolunate fusion, would be an appropriate treatment [27, 28].

The limitations of this study are the small sample size and the lack of long-term follow-up results, and no comparisons could be made among the patients since only arthroscopic thermal shrinkage or open suture tensioning was used. The results were based on the stability test, functional evaluation, and patient-reported outcomes, without a series of detectable imaging evaluations, which could be a limitation in the evaluation.

## Conclusions

Arthroscopic thermal shrinkage and arthroscopic-assisted landmark setting for open suture tensioning may be efficient and effective in the management of dynamic radiocarpal instability. However, further studies are needed to evaluate the results of this method by including a high number of cases and a long follow-up period.

## Data Availability

The datasets used and/or analyzed during the current study are available from the corresponding author on reasonable request.

## Abbreviations

DASH: Disabilities of the Arm, Shoulder and Hand questionnaire
CIND: Carpal instability nondissociative
CT: Computed tomography
FCR: Flexor carpi radialis
LRL: Long radiolunate
ROM: Range of motion
SL: Scapholunate
RSC: Radioscaphocapitate
VAS: Visual analogue scale
